# Mitochondrial metabolic reprogramming, quality control, and intercellular transfer in regulating macrophage plasticity

**DOI:** 10.3389/fphys.2026.1721230

**Published:** 2026-05-01

**Authors:** Guanheng He, Qian Sun

**Affiliations:** Department of Anesthesiology, Renmin Hospital of Wuhan University, Wuhan, China

**Keywords:** intercellular mitochondrial transfer, macrophage polarization, metabolic reprogramming, mitochondria, mitophagy

## Abstract

Macrophage functional plasticity is intrinsically linked to metabolic reprogramming, including mitochondrial function, substrate utilization, and redox signaling. In response to hypoxia, infection, or tissue injury, macrophages rely on mitochondria not only for energy provision but, critically, for metabolic intermediates and reactive oxygen species (ROS) that serve as signaling molecules to guide gene expression reprogramming. While macrophage activation exists along a continuous spectrum, this review summarizes the distinct metabolic paradigms characterizing the classical M1-like (glycolysis-dominant) and M2-like (oxidative phosphorylation, OXPHOS-dominant) extremes, highlighting the molecular mechanisms where metabolic events—specifically tricarboxylic acid (TCA) cycle truncation and succinate accumulation—drive inflammatory polarization. Furthermore, we discuss the role of mitochondrial quality control, particularly dynamics and mitophagy, in maintaining macrophage homeostasis. Notably, recent evidence identifies “intercellular mitochondrial transfer” as a novel mode of immune microenvironment regulation, enabling damaged macrophages to restore function by acquiring exogenous mitochondria. A deeper understanding of these mechanisms offers new intervention targets for metabolic immunotherapy in sepsis, cancer, and chronic inflammatory diseases. Importantly, we emphasize that many of these metabolic and mitochondrial regulatory mechanisms are highly context-dependent, varying significantly across different tissues and disease microenvironments.

## 1 Introduction

Macrophages act as the first line of defense in innate immunity and play indispensable roles in tissue development, metabolic homeostasis, and injury repair (Murray et al., 2014; Wynn and Vannella, 2016; Mosser et al., 2021). Distributed widely across tissues, these cells exhibit high functional plasticity, dynamically switching between homeostatic maintenance and rapid responses to danger signals (Orecchioni et al., 2019; Luo et al., 2024). Classically, macrophages are categorized into two polarized states based on distinct activation signals: M1 (classically activated) macrophages, induced by lipopolysaccharide (LPS) or Th1 cytokines (e.g., IFN-γ), execute bactericidal functions via the release of proinflammatory cytokines and ROS; conversely, M2 (alternatively activated) macrophages, stimulated by Th2 cytokines (e.g., IL-4/IL-13), mediate anti-inflammatory responses and tissue repair (Kelly and O’Neill, 2015; O’Neill et al., 2016). Although *in vivo* macrophage phenotypes represent a complex continuum, elucidating the mechanisms driving these phenotypic transitions remains a central theme in immunology (Medzhitov, 2008; Mosser et al., 2021).

Historically, research on macrophage polarization focused on membrane receptor signaling and transcriptional cascades, viewing intracellular metabolic shifts merely as passive bystanders. However, with the rise of immunometabolism, accumulating evidence suggests that metabolic reprogramming is a prerequisite for immune cell fate decisions (Van den Bossche et al., 2017; Jones and Divakaruni, 2020). For instance, M1 polarization is accompanied by a robust upregulation of glycolysis (resembling the Warburg effect) and suppression of mitochondrial respiration (Palsson-McDermott and O’Neill, 2013; Li et al., 2022). This metabolic shift not only provides rapid energy but also accumulates specific metabolites (e.g., succinate, citrate) that directly regulate key transcription factors such as HIF-1α (Wculek et al., 2022; Li et al., 2025). In contrast, M2 macrophages rely heavily on mitochondrial oxidative phosphorylation (OXPHOS) and fatty acid oxidation (FAO) to sustain their long-term reparative functions (Derlet et al., 2016; Sun et al., 2022).

Beyond metabolic pathway rewiring, the physical integrity of mitochondria serves as an upstream checkpoint for macrophage function. Mitochondria are not static organelles; they undergo continuous fusion and fission dynamics and rely on precise mitophagy mechanisms to eliminate damaged components (Pickles et al., 2018; Rambold and Pearce, 2018). Dysregulation of this quality control system leads to the release of ROS or mitochondrial DNA (mtDNA), which act as endogenous danger signals to persistently activate inflammasome pathways (Zhang et al., 2010; Liu et al., 2021). Furthermore, recent perspectives have expanded to the intercellular level, revealing that mitochondria can be transferred between cells via tunneling nanotubes or microvesicles (Shanmughapriya et al., 2020; Valenti et al., 2021). This “transcellular organelle transplantation” offers a novel paradigm for the metabolic rescue of impaired macrophages. This review systematically explores the core role of mitochondria in regulating macrophage plasticity through three progressive dimensions: metabolic reprogramming, organelle quality control, and intercellular mitochondrial transfer, providing new insights for treating related immune diseases (McCully et al., 2009). Rather than representing independent regulatory layers, these mitochondrial processes appear to be tightly interwoven, and dissecting their relative contribution to macrophage plasticity remains an ongoing challenge.

## 2 Metabolic reprogramming: the driving engine of macrophage polarization

Mitochondria support macrophage function by generating ATP and biosynthetic intermediates through oxidative phosphorylation (OXPHOS), fatty-acid oxidation (FAO), and amino-acid metabolism. It is now widely accepted that dynamic shifts in these metabolic pathways are not merely passive adaptations to energy demands, but rather active drivers that dictate specific immune functions and phenotypic transitions (Burcelin and Pomié, 2016).

### 2.1 Glycolytic shift, pentose phosphate pathway, and TCA cycle truncation in M1 polarization

**Figure 1:**
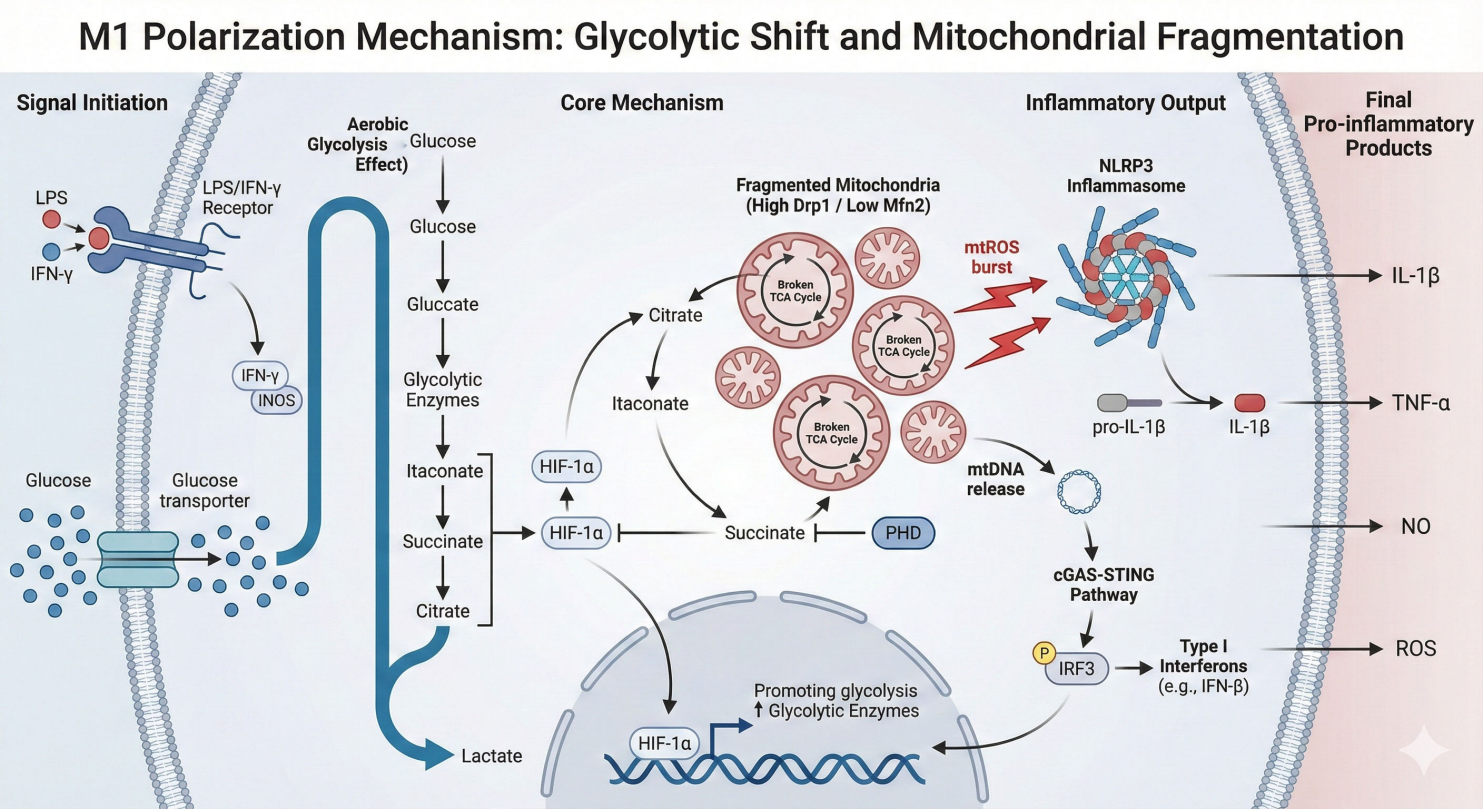
The interplay between metabolic reprogramming and mitochondrial dysfunction may drive M1 macrophage polarization.

Upon stimulation with proinflammatory signals (LPS or IFN-γ), macrophages undergo a metabolic phenotypic shift analogous to the “Warburg effect” observed in tumor cells (Ge et al., 2020; Daverio et al., 2023). However, the biological imperative for this shift fundamentally differs from that in malignancy. While cancer cells rely on aerobic glycolysis to rapidly generate biomass and metabolic intermediates required for uncontrolled mitotic division, mature tissue macrophages are largely non-proliferative (Palsson-McDermott and O’Neill, 2013). Instead, macrophages repurpose the Warburg effect to fuel an inflammatory profile. This rapid glycolytic flux not only ensures a rapid supply of ATP for phagocytosis but also allows for the strategic accumulation of specific TCA cycle intermediates (such as succinate and citrate) that function as signaling molecules to drive proinflammatory gene transcription. While mitochondria do not become completely inactive, cellular glucose uptake increases significantly (Freemerman et al., 2019). Through the upregulation of GLUT1 transporters and key enzymes such as hexokinase (Freemerman et al., 2019), metabolic flux is primarily diverted toward glycolysis to rapidly generate ATP and lactate (Ge et al., 2020; Li et al., 2022). Recent NMR studies confirm that this Warburg-associated acidification can repress fermentation independently of lactate, highlighting the complexity of this shift. Furthermore, the pentose phosphate pathway is also upregulated (Ge et al., 2020) to support nucleotide synthesis and redox homeostasis during this high-demand state. These metabolic alterations collectively establish the proinflammatory phenotype of M1 macrophages and are schematically illustrated in **Figure 1**.

Crucially, the tricarboxylic acid (TCA) cycle in M1 macrophages undergoes fragmentation at two specific checkpoints, leading to the aberrant accumulation of intermediate metabolites. First, isocitrate dehydrogenase (IDH) activity is suppressed, increasing the production of itaconate. Itaconate not only possesses direct antimicrobial properties but also inhibits succinate dehydrogenase (SDH) (Peace and O’Neill, 2022). Second, the blockade of electron transport at SDH leads to the massive accumulation of succinate. Extensive research demonstrates that accumulated succinate inhibits prolyl hydroxylase (PHD) activity, thereby preventing the degradation of HIF-1α (Tannahill et al., 2013; Huang et al., 2022). Stabilized HIF-1α subsequently translocates to the nucleus to initiate the transcription of proinflammatory genes, including IL-1β. This mechanism illustrates how mitochondrial metabolites directly function as signaling molecules to drive inflammatory responses (Tannahill et al., 2013). As shown in **Figure 1**, metabolic reprogramming in M1 macrophages is closely associated with mitochondrial fragmentation, enhanced glycolysis, and increased mtROS production, which collectively amplify pro-inflammatory signaling pathways. Notably, most evidence supporting this model derives from acute *in vitro* stimulation, and whether sustained succinate accumulation uniformly may drive inflammatory polarization across tissue-resident macrophages remains less clear.

### 2.2 OXPHOS and anaplerosis in M2 polarization

In stark contrast to M1 cells, M2 macrophages (induced by IL-4/IL-13) are responsible for tissue repair and inflammation resolution—processes requiring sustained and efficient energy support (Braga et al., 2015; Yu et al., 2022). Consequently, M2 cells maintain an intact TCA cycle and rely heavily on FAO and OXPHOS (Im et al., 2011). To sustain the continuous operation of the TCA cycle, M2 cells significantly enhance glutaminolysis as a source of carbon anaplerosis (Artyomov and Van Den Bossche, 2020; Kieler et al., 2021).

During this process, cells upregulate glutamine transporters and glutamine synthetase (GS), converting glutamine into α-ketoglutarate (α-KG) to enter the TCA cycle. α-KG serves not only as a metabolic intermediate but also as a cofactor for epigenetic enzymes such as Jmjd3, promoting histone demethylation of M2 marker genes (e.g., *Arg1*), thereby epigenetically consolidating the M2 phenotype (Kieler et al., 2021). Although recent studies suggest metabolic heterogeneity among M2 macrophages of different tissue origins (e.g., peritoneal vs. bone marrow-derived), an OXPHOS-dominant metabolic network remains the most defining feature distinguishing them from the M1 phenotype (Freemerman et al., 2019; Doblado et al., 2021; Wculek et al., 2022). However, emerging evidence suggests that the metabolic requirements of M2-like macrophages are highly context-dependent, varying substantially across tissue niches and disease settings (Artyomov and Van Den Bossche, 2020; Wculek et al., 2022).

## 3 Mitochondrial quality control in M1 and M2 macrophage polarization

Beyond metabolic pathway alterations, the physical integrity of mitochondria—governed by dynamics (fusion/fission) and autophagic clearance—constitutes an upstream checkpoint regulating macrophage polarization (Doblado et al., 2021).

### 3.1 Mitochondrial fission and inflammatory signal amplification

In M1 macrophages, mitochondrial fragmentation is closely associated with inflammatory activation, whereas in M2 macrophages, mitochondrial integrity is generally preserved to support anti-inflammatory functions. The dynamic shaping of mitochondrial morphology is a direct manifestation of macrophage adaptation to microenvironmental stress. In the early stages of M1 polarization, mitochondria undergo marked fragmentation, a process primarily mediated by the dynamin-related protein Drp1 (Rowland and Voeltz, 2012). While this fragmented architecture reduces OXPHOS efficiency, it is accompanied by a burst of mitochondrial ROS (mtROS) generation (Waypa et al., 2016; Aklima et al., 2021).

As introduced previously, mitochondrial dysfunction amplifies inflammatory signaling; specifically, electron leakage generates mtROS, acting as an essential trigger for NLRP3 inflammasome assembly (Selivanov et al., 2008). Furthermore, severe mitochondrial damage alters membrane permeability, causing the release of mtDNA into the cytosol (West et al., 2011). As an endogenous pathogen-associated molecular pattern (DAMP), cytosolic mtDNA is recognized by the DNA sensor cGAS, subsequently activating the STING-IRF3 axis to induce type I interferon secretion (West et al., 2011). Thus, excessive mitochondrial fission and the resultant leakage of ROS/mtDNA establish a **feed-forward loop** that sustains the M1 inflammatory phenotype (Bedard and Krause, 2007; Zhou et al., 2011; Zhou et al., 2023; Yan et al., 2024; Sun et al., 2025). These processes further reinforce the inflammatory signaling network described earlier (as illustrated in **Figure 1**).

### 3.2 Mitophagy: the guardian of the M2 phenotype

In contrast to M1 macrophages, M2 macrophages rely on efficient mitochondrial quality control mechanisms to maintain metabolic stability. Conversely, M2 macrophages must maintain low ROS levels to prevent tissue damage, a capability largely attributed to efficient mitophagy (Bussi et al., 2022; Chen et al., 2025). This process is primarily mediated by the PINK1-Parkin pathway: when mitochondrial damage leads to a drop in membrane potential, PINK1 kinase accumulates on the outer mitochondrial membrane, recruiting and phosphorylating the ubiquitin ligase Parkin (Spratt et al., 2013; Sun et al., 2018). These tags damaged mitochondria with ubiquitin, ultimately mediating their lysosomal degradation (Ashrafi and Schwarz, 2013; Zhao et al., 2020).

Experimental evidence shows that blocking autophagic flux with drugs (e.g., 3-methyladenine) or specifically knocking out autophagy-related genes leads to the accumulation of dysfunctional mitochondria within macrophages (Sun et al., 2018). These “garbage” organelles spontaneously release ROS and activate the NLRP3 inflammasome, causing macrophages to exhibit a “mixed” inflammatory phenotype even under IL-4 induction, preventing successful M2 transition (Herzig and Shaw, 2018; Yang et al., 2025). Additionally, the energy sensor AMPK plays a dual role in this context: it promotes mitochondrial biogenesis while synergistically initiating autophagy, ensuring that M2 cells consistently maintain a high-quality mitochondrial network (Canugovi et al., 2010). It should be noted that PINK1–Parkin–dependent mitophagy may not represent the sole pathway governing mitochondrial quality control in macrophages, and alternative mechanisms may compensate under certain inflammatory conditions. For instance, emerging evidence indicates that macrophages may also utilize mitochondria-derived vesicles (MDVs) to selectively extrude damaged mitochondrial components, offering a complementary quality control layer before whole-organelle degradation is required (Pickles et al., 2018).

Upon LPS and IFN-γ stimulation, macrophages undergo a glycolytic shift (Warburg effect), leading to rapid lactate production. Concurrently, the TCA cycle is truncated at isocitrate dehydrogenase and succinate dehydrogenase (SDH), resulting in the accumulation of itaconate and succinate. Accumulated succinate stabilizes HIF-1α to promote proinflammatory gene expression (e.g., IL-1β). Simultaneously, mitochondrial fragmentation (driven by Drp1) induces a burst of mitochondrial ROS (mtROS) and the release of mtDNA into the cytosol. These mitochondrial danger signals further amplify the inflammatory output by activating the NLRP3 inflammasome and the cGAS-STING-IRF3 pathway, respectively.

## 4 Intercellular mitochondrial transfer: an emerging paradigm for phenotypic remodeling

While classical metabolic regulation is confined within the cell, recent studies reveal that “intercellular mitochondrial transfer” functions as a critical transcellular communication mechanism in remodeling the immune microenvironment (Derlet et al., 2016). This transfer is not merely a substance exchange but acts as a modulatory signal that can significantly influence the polarization fate of recipient macrophages, though the precise deterministic nature of these mechanisms remains under active investigation.

### 4.1 Mesenchymal stem cell-mediated metabolic rescue

While M1 macrophages are typically characterized by metabolic dysfunction, M2-like phenotypes can be restored through mitochondrial transfer under certain conditions. In models of tissue injury or sepsis, mesenchymal stem cells (MSCs) exhibit potent immunomodulatory capabilities (Morrison et al., 2017), with a core mechanism being the transfer of functional mitochondria to impaired macrophages. This process relies primarily on tunneling nanotubes (TNTs) constructed by the actin cytoskeleton or the release of mitochondria-containing extracellular vesicles (EVs) (, Sun et al., 2013; Morrison et al., 2017; Main et al., 2023).

**Figure 2:**
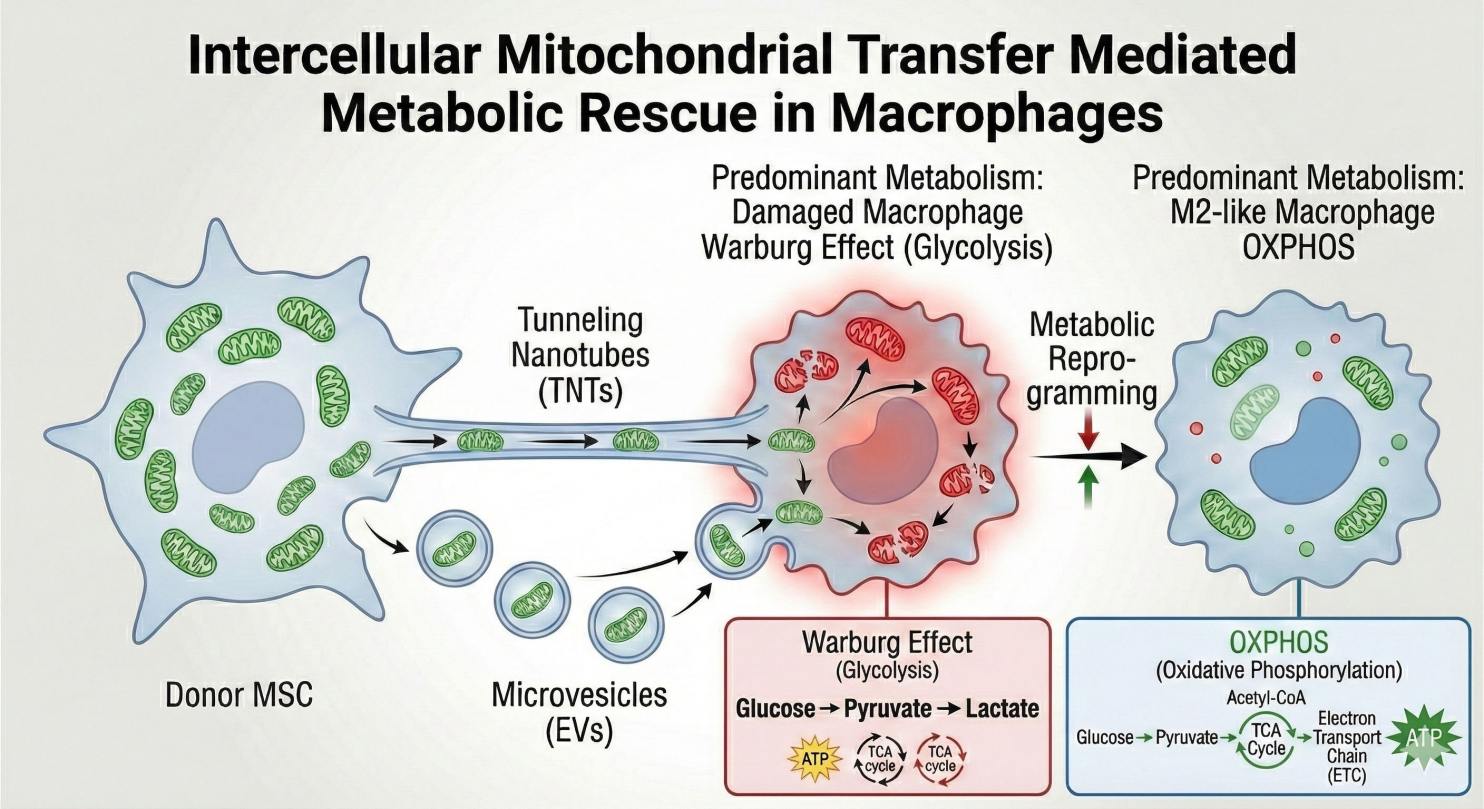
Intercellular mitochondrial transfer promotes metabolic rescue and M2 polarization.

Research confirms that when highly inflammatory M1-like macrophages capture exogenous healthy mitochondria, their intracellular metabolic landscape is rapidly reversed (as illustrated in **Figure 2**, mitochondrial transfer can restore oxidative phosphorylation and reshape the metabolic and functional phenotype of recipient macrophages, although these effects are highly context-dependent): the integration of exogenous mitochondria restores OXPHOS levels in recipient cells and reduces reliance on glycolysis. This metabolic reprogramming transcriptionally suppresses proinflammatory gene expression and induces the upregulation of M2 markers. This phenomenon suggests that the proinflammatory phenotype of macrophages is largely a consequence of endogenous mitochondrial dysfunction, and exogenous organelle supplementation may be sufficient to overcome metabolic barriers under specific conditions to partially overcome metabolic barriers and restart anti-inflammatory and reparative programs (Morrison et al., 2017) (as illustrated in **Figure 2**).

### 4.2 Mitochondrial reprogramming in ischemia-reperfusion injury

To further illustrate the clinical relevance of these mechanisms, ischemia-reperfusion injury (IRI)—a profound challenge in solid organ transplantation and acute ischemic events—serves as an excellent model (Lee et al., 2011). During the ischemic phase, severe hypoxia compromises mitochondrial OXPHOS, forcing tissue-resident macrophages to rely on anaerobic glycolysis. Upon reperfusion, the sudden influx of oxygen rapidly interacts with the stalled electron transport chain, generating a massive surge of mtROS. This ROS burst, coupled with succinate oxidation, acts as a potent trigger for M1 polarization, driving extensive secondary tissue damage (Chouchani et al., 2014). Recent preclinical studies suggest that targeting macrophage mitochondrial dynamics or facilitating intercellular mitochondrial transfer could mitigate this hyper-inflammatory response, accelerating the transition towards an M2-like reparative phase and ameliorating fibrotic outcomes following acute injury (Mao et al., 2022). These processes are conceptually consistent with the mitochondrial rescue mechanisms summarized in **Figure 2**.

### 4.3 Pathological interactions in the tumor microenvironment

In the complex landscape of the tumor microenvironment (TME), the machinery of mitochondrial transfer is frequently subverted to facilitate tumor progression rather than homeostatic recovery (Liu et al., 2025). Malignant cells expel various mitochondrial components—including mitochondrial DNA (mtDNA), cytochrome c, and formylated peptides—into the extracellular milieu. These molecules act as Damage-Associated Molecular Patterns (DAMPs), which are defined as endogenous danger signals typically sequestered within healthy cells that, when released into the extracellular space during stress or necrosis, activate innate immune sensors via pattern recognition receptors.

The interaction between these mitochondrial DAMPs and resident macrophages is strongly associated with tumor progression, potentially serving as a driver of malignancy in specific contexts (Chun et al., 2025; Liu et al., 2025). For instance, mtDNA released into the TME can be recognized by the DNA sensor TLR9, subsequently triggering chronic NF-κB signaling activation within macrophages. Rather than initiating an effective anti-tumor response, this pathological activation often promotes a metabolic and transcriptional shift toward an immunosuppressive, M2-like phenotype, commonly referred to as tumor-associated macrophages (TAMs). These TAMs facilitate an environment conducive to cancer cell proliferation, angiogenesis, and invasion while actively suppressing the recruitment and cytotoxic activity of T cells (Chun et al., 2025).

Furthermore, horizontal mitochondrial transfer can occur between cancer cells and stromal cells, or among cancer cells themselves, to enhance their overall bioenergetic fitness and resistance to chemotherapy. Consequently, the functional outcome of mitochondrial transfer in the TME is profoundly context-dependent. Unlike the “metabolic rescue” observed with mesenchymal stem cells, mitochondrial crosstalk in the TME represents a pathological hijacking of metabolic pathways that recalibrates the immune response to support tumor survival instead of tissue resolution (Ishino and Togashi, 2025).

Mechanism of Transfer: Healthy donor cells, such as Mesenchymal Stem Cells (MSCs), donate functional mitochondria (green) to damaged, pro-inflammatory (M1-like) macrophages via actin-based tunneling nanotubes (TNTs) or extracellular vesicles (EVs). Metabolic Reprogramming: The integration of exogenous healthy mitochondria triggers a fundamental shift in the recipient’s metabolic landscape. Before Rescue (Damaged Macrophage): The predominant metabolism is the Warburg Effect (Aerobic Glycolysis), where glucose is rapidly converted to pyruvate and then to lactate, bypassing a fragmented TCA cycle, and produces rapid but inefficient ATP. After Rescue (M2-like Macrophage): Functional mitochondria restore Oxidative Phosphorylation (OXPHOS). Pyruvate is converted to Acetyl-CoA to fuel an intact TCA Cycle, driving the Electron Transport Chain (ETC) for efficient and sustained ATP production. Functional Outcome: This “metabolic rescue” suppresses pro-inflammatory gene expression and induces the transition toward an anti-inflammatory, reparative M2-like phenotype, effectively restarting the tissue repair program.

## 5 Conclusion and perspectives

In summary, the role of mitochondria in macrophage biology extends far beyond the traditional definition of “powerhouses.” From intracellular metabolic pathway rewiring (e.g., glycolysis and TCA cycle truncation) to organelle-level quality surveillance (dynamics and autophagy), and finally to intercellular organelle transfer, mechanisms across these three dimensions collectively weave the regulatory network of macrophage polarization (Laird et al., 2024; Sun et al., 2025).

Despite rapid progress, the field faces significant challenges. First, most current *in vitro* experiments utilize high-glucose, high-oxygen culture media, which differ vastly from the hypoxic, nutrient-poor microenvironments found *in vivo* (particularly in infection or tumor sites), potentially biasing experimental results (Toor et al., 2021; Thome et al., 2022). Second, the specific molecular recognition mechanisms of mitochondrial transfer—how recipient cells specifically “uptake” rather than “digest” exogenous mitochondria—remain incompletely understood (Kuznetsov et al., 2019; Huang et al., 2025). Future research should combine single-cell metabolomics with *in vivo* lineage tracing technologies to further elucidate the spatiotemporal dynamics of mitochondrial quality control in chronic inflammation and tumor progression (Artyomov and Van Den Bossche, 2020; Bouhamida et al., 2022). This will not only deepen our understanding of immunometabolism but also provide a theoretical basis for developing precision immunotherapies that target mitochondrial restoration or block specific metabolic pathways.

Crucially, the context-dependent nature of both metabolic reprogramming and intercellular mitochondrial transfer remains a major frontier. Mechanisms observed in acute injury models may not entirely translate to chronic conditions or specific tumor niches, necessitating highly contextualized future studies. Future studies are required to clarify the causal relationships and context-specific variability underlying mitochondrial regulation in macrophage biology.
